# A Unique 3D Nitrogen-Doped Carbon Composite as High-Performance Oxygen Reduction Catalyst

**DOI:** 10.3390/ma10080921

**Published:** 2017-08-09

**Authors:** Ramesh Karunagaran, Tran Thanh Tung, Cameron Shearer, Diana Tran, Campbell Coghlan, Christian Doonan, Dusan Losic

**Affiliations:** 1 School of Chemical Engineering, University of Adelaide, SA 5005, Australia; ramesh.karunagaran@adelaide.edu.au (R.K.); tran.tung@adelaide.edu.au (T.T.T.); diana.tran@adelaide.edu.au (D.T.); 2 School of Chemistry, University of Adelaide, SA 5005, Australia; cam.coghlan@adelaide.edu.au; 3 School of Chemical and Physical Sciences, Flinders University, SA 5005, Australia; Cameron.Shearer@flinders.edu.au

**Keywords:** N-doped carbon spheres, N-doped carbon nanotubes, ORR, hybrid, catalysts

## Abstract

The synthesis and properties of an oxygen reduction catalyst based on a unique 3-dimensional (3D) nitrogen doped (N-doped) carbon composite are described. The composite material is synthesised via a two-step hydrothermal and pyrolysis method using bio-source low-cost materials of galactose and melamine. Firstly, the use of iron salts and galactose to hydrothermally produceiron oxide (Fe_2_O_3_) magnetic nanoparticle clusters embedded carbon spheres. Secondly, magnetic nanoparticles diffused out of the carbon sphere when pyrolysed in the presence of melamine as nitrogen precursor. Interestingly, many of these nanoparticles, as catalyst-grown carbon nanotubes (CNTs), resulted in the formation of N-doped CNTs and N-doped carbon spheres under the decomposition of carbon and a nitrogen environment. The composite material consists of integrated N-doped carbon microspheres and CNTs show high ORR activity through a predominantly four-electron pathway.

## 1. Introduction

Rising energy demands and the depletion of non-renewable fossil fuels have generated significant interest in renewable energy sources such as solar, wind, and geothermal [1]. Among alternate energy devices currently being considered, particularly for mobile applications, fuel cells have been widely investigated due to their theoretically high efficiencies and low carbon footprint [2]. In a hydrogen fuel cell O_2_ and H_2_ are converted into electricity and water. This process involves reducing O_2_ at the cathode, a reaction typically carried out by expensive platinum-based catalysts. Thus, the commercial viability of hydrogen fuel cells requires the development of low-cost and efficient oxygen reduction reaction (ORR) catalysts [3].

Recent research efforts have focused on the development of alternative catalysts that can be synthesised from readily available starting materials while also overcoming the drawbacks of the Pt catalysts [4]. Several materials, such as transition metals, spinel catalysts, perovskite catalysts, metal carbon hybrid catalysts and non-metal doped catalysts, have been used as an alternative for Pt catalysts [5]. Among the possible alternatives, doped carbon nanomaterials (i.e., graphitic carbons) have been recognised as promising materials due to their high catalytic activity and low production cost [6]. Gong et al. [7] first reported that non-metal N-doped carbon materials can show outstanding ORR performance. Since then, doping graphitic carbons with heteroatoms (e.g., nitrogen, boron, sulphur, phosphorous) has been used to increase ORR catalytic activity [8]. Transition metals such as iron and cobalt can facilitate both the incorporation of the heteroatoms and the formation of active sites within the graphitic carbon structure [9]. The primary approach to synthesizing nitrogen-doped (N-doped) mesoporous carbon materials with high ORR activity is via direct gas sources or pyrolysis of nitrogen-containing compounds [10,11,12,13]. Among these N-doped mesoporous materials, carbon nanospheres have shown high activity for ORR catalysis [14,15]. The reason has been attributed to the increased surface area and hierarchical complexity of nanosphere morphologies [16]. However, multiple processing steps and the requirement of hazardous chemicals for synthesis have hindered the practical applications of these materials.

**Scheme 1:**
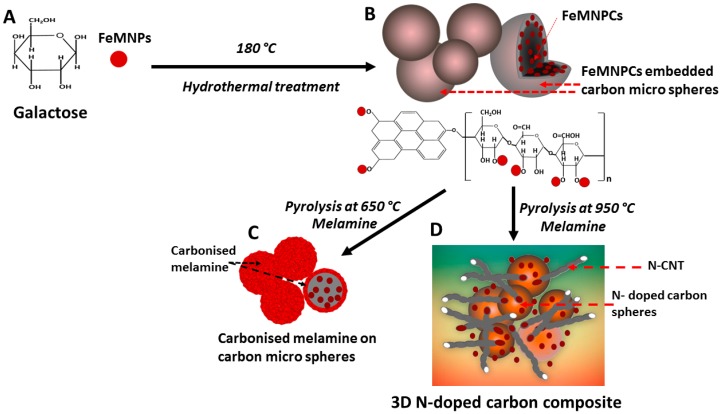
Schematic for the synthesis of the 3D N-doped carbon composites with combined N-doped microspheres and N-CNT. (**A**) Galactose and FeMNPs dispersed in water; (**B**) carbon microsphere formation with FeMNPC embedded (GAL-Fe-HT); (**C**) carbonised melamine associated with microspheres at 650 °C; (**D**) carbon nanotubes are formed from the diffused FeMNPC catalysts, forming the composite material (GAL-Fe-N).

To address these synthetic issues, unique 3D N-doped carbon composites that combine both spherical and nanotubular structures were synthesised in this work. These materials were produced using a cost-effective, environmentally-friendly and scalable method from a readily available biosource of monosaccharide (galactose) and melamine by using a two-step hydrothermal and pyrolysis method (Scheme 1). Iron oxide (Fe_2_O_3_) magnetic nanoparticle clusters (FeMNPCs) were introduced to initiate the N-doping of the material [17]. In fact, transition metals such as Fe serve to facilitate N incorporation into graphitic structures under pyrolysis [17]. The FeMNPCs (with sizes of 40–80 nm) are encapsulated within carbon microspheres formed from the carbonisation of galactose-based polysaccharide. The purified samples were subsequetly introduced to the pyrolysis of melamine at 650 °C as an N precursor, forming a carbonised layer around the microspheres, in which ratios of melamine to carbon were chosen based on previous research [18,19]. Finally, heating at 950 °C causes decomposition of the microsphere, followed by diffusion of FeMNPCs out of the carbon spheres to catalyse N-CNTs formation. Baker et al. [20] and Ozkan and co-workers [21] previously reported that metal particles can catalyse the growth of carbon fibres. The ORR catalytic properties of the as-prepared composite materials were evaluated by electrochemical characterisation, and their structural and chemical composition, as well as on the basis of their reproducibility.

## 2. Materials and Methods

### 2.1. Materials

D-(+)-Galactose (Sigma Aldrich, purity > 98.5%), iron (II) chloride tetra hydrate (FeCl_2_·4H_2_O) (Sigma Aldrich, St. Louis, MO, USA), iron (III) chloride hexahydrate (FeCl_3_·6H_2_O) (Chem Supply, Gillman, SA, Australia), hydrochloric acid (HCl) (Chem Supply, Gillman, SA, Australia), ammonia (Chem Supply, Gillman, SA, Australia), melamine (Sigma Aldrich, St. Louis, MO, USA), and platinum standard catalyst (20 wt.% Vulcan XC-72) were used as purchased.

### 2.2. Methods

#### 2.2.1. Synthesis of Maghemite Nanoparticles

Maghemite nanoparticles were synthesised according to a previously reported method [22]. Briefly, FeCl_2_·4H_2_O (39.76 g) and FeCl_3_·6H_2_O (16.29 g) were mixed with 100 mL of HCl (1 M) under stirring until all solids were dissolved. The pH of the mixture was adjusted to 9.8 using 2M ammonia solution. Then the solution was further stirred for another 2 h, followed by washing several times with DI water and ethanol with a centrifuge. Finally, the purified product was dried at 60 °C in a vacuum oven until its weight was constant.

#### 2.2.2. Synthesis of Iron Oxide Embedded Carbonaceous Spheres from Galactose

Maghemite nanoparticles (200 mg) were added to a suspension of 0.02 mole galactose in 40 mL water and mixed stirring for 30 min. The mixture was transferred to a Teflon autoclave and heated to 180 °C for 18 h. Then the product was collected, centrifuged repeatedly washed, six times with deionised water and 4 times with 0.5 M H_2_SO_4_. The product was collected and freeze dried for 24 h (referred to as GAL-Fe-HT). GAL-Fe-HT was then annealed at 950 °C under argon (Ar) for 3 h and denoted as GAL-Fe-A.

#### 2.2.3. Synthesis of N-do ped Carbon Spheres with Iron Oxide Nanoparticles

GAL-Fe-HT was mixed with melamine (1:10 *w*/*w*) and ground using a mortar and pestle, followed by annealing under the same conditions (950 °C for 3 h under Ar) and denoted as GAL-Fe-N.

#### 2.2.4. Synthesis of Carbon Spheres Without Iron Oxide Nanoparticles

A suspension of 0.02 mole galactose in 40 mL water was mixed and stirred for 30 min. The mixture was transferred to a Teflon autoclave and heated to 180 °C for 18 h. Then the product was collected, centrifuged, and washed six times with deionised water and 4 times with 0.5 M H_2_SO_4_. To dope the product with nitrogen, GAL-HT was mixed with melamine (1:10 *w*/*w*) and ground using a mortar and pestle, followed by annealing under the same conditions (950 °C for 3 h under Ar) and denoted as GAL-N.

### 2.3. Preparation of Catalytic Inks

The catalytic ink was prepared by dispersing 2 mg of catalyst in a 1 mL of 10 *v*/*v*% nafion in water. Then, 10 µL of the prepared ink was carefully deposited onto the 3 mm glassy carbon rotating disc electrode (RDE) and dried in the air. For the rotating ring disc analysis, the ink was deposited on a 4 mm glassy carbon rotating ring disc (RRDE) electrode. A similar procedure was followed for Pt/C (20 wt.% Vulcan XC-72) catalyst as the standard catalyst.

### 2.4. Characterisation

Several analytical techniques were used to characterise the synthesised products. The morphology and the structure of these samples were investigated by scanning electron microscopy (SEM) and transition electron microscopy (TEM). SEM images were collected using Quanta 450, FEI (USA) at an accelerating voltage of 10 keV. TEM measurement was carried out by using a Technai G2 Spirit, FEI (Hillsboro, OR, USA), operated at 120 keV. X-ray diffraction (XRD) was performed using a Miniflex 600, Rigaco (Tokyo, Japan) operated at 40 KV and 15 mA in the range of 2θ = 10–70° at a scan rate of 10°/min. Fourier transform infrared (FTIR) spectroscopy was conducted using a spectrum 100, Perkin Elmer, (Shelton, CT, USA). Raman analysis was done using a LabRAM Evolution, Horiba Jvon (Japan) with a laser excitation wavelength of 532 nm. Gas adsorption isotherms were conducted using a Micromeritics 3-Flex or ASAP2020 analyser (Micro metrics Instruments Corporation, Norcross, GA, USA). BrunauerEmmett—Teller (BET) surface area and pore size distribution were calculated using software on the Micromeritics 3-Flex or ASAP 2020 analyser. X-ray photo electron spectroscopy (XPS) was conducted on a SPECS instrument (Berlin, Germany). All XPS measurements were performed using a non-monochromatic Mg source operated at 200 W. High resolution XP spectra were collected using a pass energy of 10 eV for C 1s and 20 eV for N1s.

#### Electrochemical Characterisation

The ORR reactions were conducted using a rotating disc (RDE) and rotating ring disc electrode (RRDE) apparatus connected to a bi potentiostat (CH 1760 C, CH Instruments Inc., (Bee Cave, TX, USA) in a standard three-electrode cell with oxygen-saturated KOH (0.1 mol L^−1^) solution. The reaction was scanned at a scan rate of 0.01 Vs^−1^ between 0 and 1.1 V. The glassy carbon electrode was used as the working electrode, while platinum and reversible hydrogen electrodes (RHE) were used as the counter and reference electrodes, respectively. RRDE voltammograms were obtained after several repeated cycles were run between 0 and 1.1 V until stable voltammograms were obtained.

## 3. Discussion

**Figure 1:**
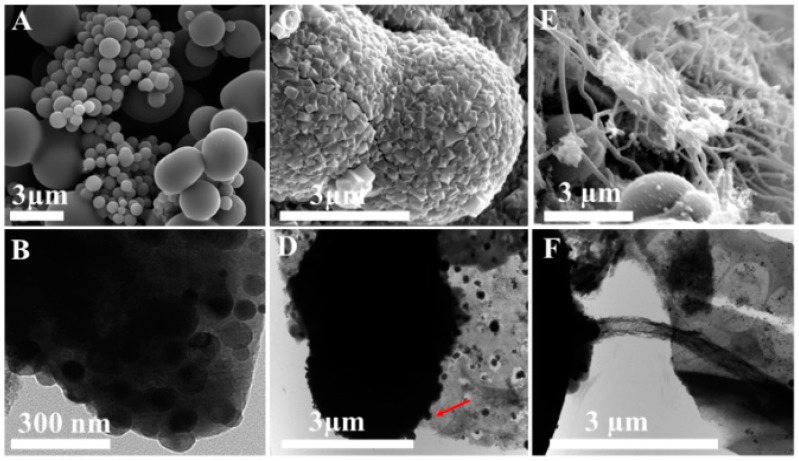
(**A**) SEM image of GAL-Fe-HT prepared using the hydrothermal process combining galactose and FeMNPCs; (**B**) TEM image confirming interior spheres were embedded with FeMNPs; (**C**) SEM image of GAL-Fe-HT pyrolysed at 650 °C with melamine; (**D**) TEM image of diffusion of FeMNPs from the sphere pyrolysed at 950 °C with melamine (red arrow FeMNPC); (**E**) SEM image of final composite material (GAL-Fe-N) with integrated N-doped carbon microspheres and N-CNT; and (**F**) TEM image of CNT forming from the FeMNPC within the sphere.

The morphology of the prepared composite materials was investigated using SEM. Figure 1A shows smooth carbon microspheres of hydrothermally reduced galactose with FeMNPCs (GAL-Fe-HT) with sizes ranging from 1 to 6 µm. The presence of FeMNPs encapsulated within the sphere was confirmed by TEM (Figure 1B). The FeMNPCs are encapsulated within the carbon sphere through coulombic interactions formed between surface functional groups (i.e., OH, C=O) of galactose and Fe_2_O_3_ [23]. N-doping was introduced by pyrolysing the GAL-Fe-HT with melamine at 950 °C. SEM images were analysed at the intermediate stages of pyrolysis at 650 and 950 °C. At the intermediate stage, the microspheres undergo a significant shift in surface chemistry as the melamine becomes carbonised and attaches to the spheres (Figure 1C). Close inspection of the TEM image (Figure 1D) shows that subsequent heating from 650 to 950 °C causes the carbonised melamine surrounding the spheres to decompose causing surface damage to the carbon sphere allowing the FeMNPCs contained within the spheres to be released. Energy dispersive X-ray (EDX) analysis conducted on these particles (Figure 1D) revealed an iron content of 7.52 wt.%, confirming the presence of the iron oxide particles (Supplementary Information (SI)). We note that a novel morphological change occurred as the FeMNPs were released. Figure 1E shows that the C and N precursors from pyrolysed melamine formed N-CNT along with N-doped carbon spheres, giving rise to a unique 3D architecture. EDX analysis conducted on the carbon microspheres and CNT showed 2.55 and 2.77 At.% (atomic percentage) of N, confirming diffusion of N precursors into both carbon spheres and CNT. To verify this hypothesis, GAL-Fe-HT was pyrolysed without melamine at 950 °C (GAL-Fe-A). Separately, galactose carbon spheres (GAL) were synthesised without iron oxide nanoparticles and pyrolysed at 950 °C with melamine (GAL-N). The SEM of the GAL-Fe-A or GAL-N (SI) displayed only carbon spheres. This confirms that the formation of the N-CNT resulted from the iron oxide particles.

**Figure 2:**
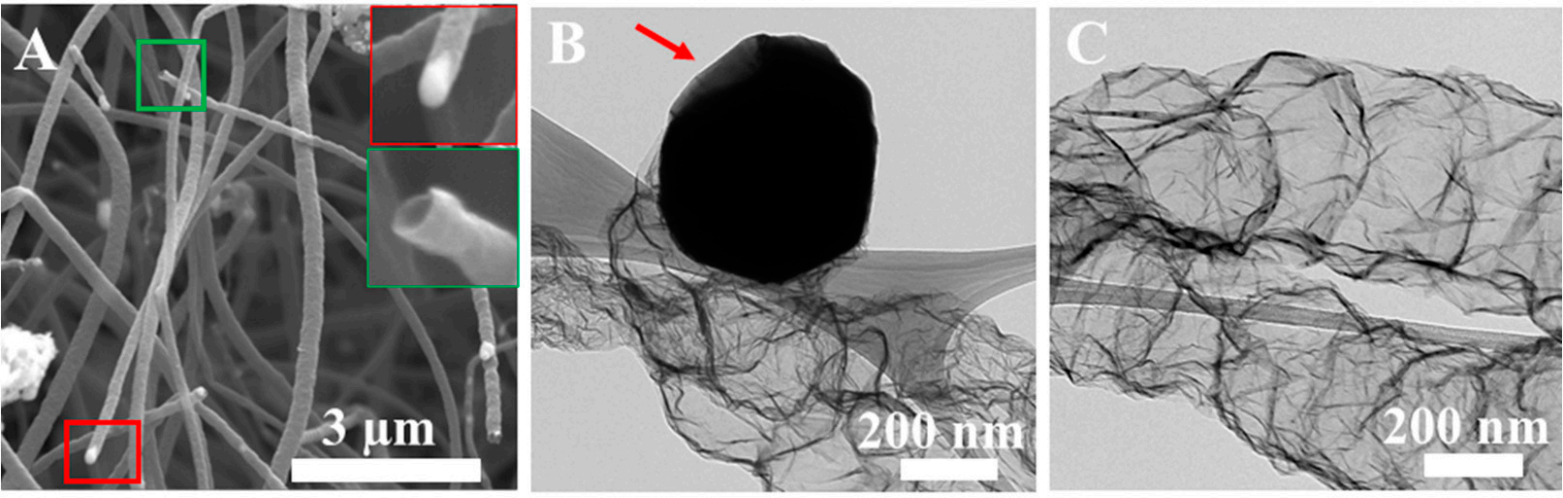
SEM image of (**A**) carbon nanofibers; the inset red and green outlines are the FeMNPCs attached to the tip of the tube and the cross section of a hollow nanotube, respectively; TEM image of (**B**) formation of a CNT from a FeMNPC, red arrow shows the utilised area of carbon precursors and (**C**) hollow corrugated CNT.

The CNT structure was analysed further using SEM and TEM. SEM of the CNTs (Figure 2A) shows both capped and uncapped CNTs. Interestingly, EDX analysis of the capped CNT reveals a high percentage (31.86 At.%) of Fe at the tip, indicating the presence of FeMNPCs. Furthermore, the TEM images (Figure 2B,C) confirm that the FeMNPCs are located at the tips of the CNTs and possess a hollow corrugated structure containing an irregular compartmentalised morphology. When melamine is pyrolysed, the decomposition product carbon is exposed on the surface of the reduced metallic Fe particle [24]. Then, the carbon diffuses through the Fe particles and precipitates on the other side, which is relatively cooler than the exposed surface [25]. The resultant products form a thin-walled, hollow and aligned CNT with a corrugated morphology [25]. Terrones et al. [26] synthesised similar carbon nano fibers (CNF) and reported that the nitrogen precursor from the decomposed melamine is bonded to C in sp^2^ pyridine like and sp^3^ bridge-head nitrogen type in CNF. Authors postulated that the formation of a rough corrugated morphology occurs when the nitrogen content of the material is increased as a replacement of C with N during doping. Also, the curvature of CNF occurs due to the carbon vacancies formed within the predominantly hexagonal graphene network of CNF [26]. A detailed analysis of the Raman spectrum, XRD diffraction peaks, BET surface area analysis and FTIR spectrum of GAL-Fe-N are presented in the SI. XRD analysis conducted on the maghemite nanoparticles is also presented in SI.

**Figure 3:**
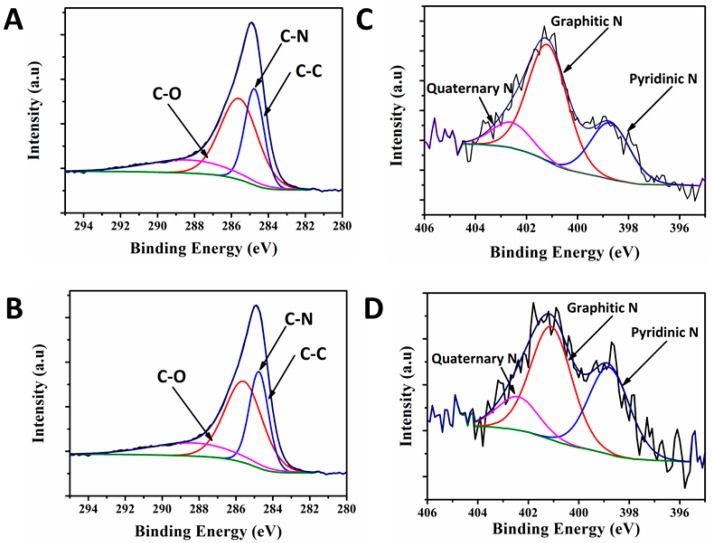
(**A**,**B**) are the XPS spectra of the deconvoluted C1s region; and (**C**,**D**) are the deconvoluted N1s region of GAL-N and GAL-Fe-N, respectively.

The surface of the GAL-N and GAL-Fe-N composite materials was probed by XPS and the data presented in Figure 3. Figure 3A,B shows that the carbon spectra include peaks at C-C (284.79 eV), C-N (285.75 eV) and C-O (287.76 eV) for GAL-N and GAL-Fe-N, respectively [27]. While the N1s XPS spectra shows three distinct peaks attributed to: pyridine-N (398.8 eV), graphitic-N (401.09 eV) and quaternary amine-N (402.42 eV) (Figure 3C,D) for both catalysts [28], pyrrolic nitrogen was not detected on these samples because at high pyrolysis temperatures (e.g., 800 °C), the pyrrolic nitrogen atom in the five-sided ring is thermally unstable and is converted into a graphitic nitrogen atom in the graphitic carbon frame-work [29]. The pyridinic N (27.80 At.%) present in GAL-N and (37.37 At.%) in GAL-Fe-N provides essential Lewis basicity to the adjacent carbon atoms, which is vital to initial oxygen uptake and for ORR catalysis [30]. However, the presence of Fe was not detected by XPS. This confirms Fe-Nx species are not present in the catalyst, which could also act as an active site [31].

**Figure 4:**
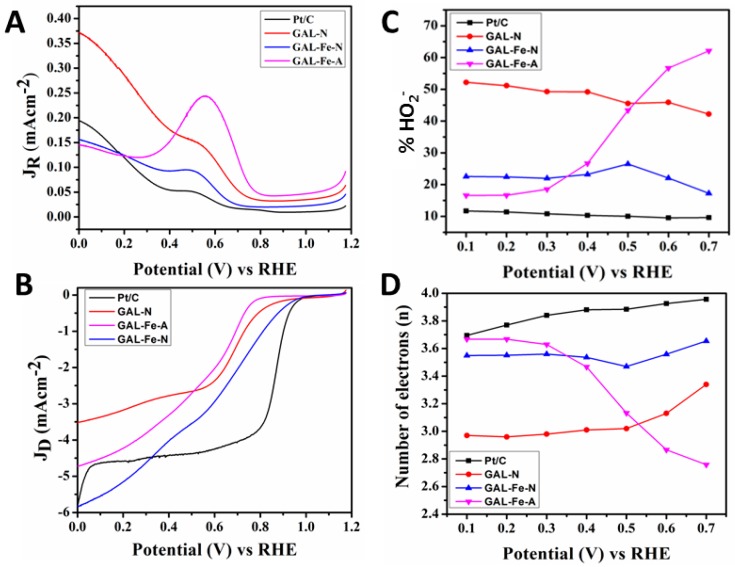
Rotating ring disc voltammograms recorded with GAL-N, GALFe-A, GALFe-N and Pt/C electrodes. (**A**) Ring current; (**B**) disc current; (**C**) HO_2_^−^ peroxide produced (%) against applied potential; and (**D**) number of total electrons transfers against applied potential.

Figure 4A,B shows the ring and the disc current of the GAL-N, GAL-Fe-A, GAL-Fe-N and Pt/C catalysts with 2000 rpm recorded at scan rate of 10 mVs^−1^, respectively Table 1 shows that the onset overpotential for GAL-Fe-N (0.29 V) is less by 160 mV and 40 mV than those of GAL-Fe-A (0.45 V) and GAL-N (0.33 V), respectively. The greater onset overpotential of GAL-Fe-A reveals that the magnetic nanoparticles present in the micro spheres did not provide enough active sites to efficiently initiate the ORR reaction. Conversely, both N-doped catalysts (GAL-N and GAL-Fe-N) provided greater catalytic activity than GAL-Fe-A catalysts and showed a lower onset overpotential when compared with GAL-Fe-A. The 40 mV lower overpotential for GAL-Fe-N when compared to GAL-N indicates that GAL-Fe-N provided more active sites for oxygen reduction than the N-doped carbon spheres in GAL-N. The half wave potential of GAL-Fe-N shifted negatively when compared to Pt/C, indicating that the reaction is carried out by mixed kinetic and diffusion mechanisms where the current is controlled by both mass transport and kinetics electron transfer [32].

The % HO_2_^−^ yield and the overall electron transfer number for the ORR reaction was calcuated using RRDE values and is shown in Figure 4C,D, respectively. The results presented in Table 1 reveal that amongst all the catalysts, GAL-Fe-N performed effectively by driving the reaction via a predominantly four-electron pathway in the potential range of 0.10–0.70 V. Within this potential range, the electron transfer number changed from 3.55 to 3.64, with a HO_2_^−^ yield of 22.44–16.96%, showing excellent ORR performance. Previous work suggests two different active sites for ORR reaction for M-N/C catalysts; M-Nx species, and N-atom-doped carbon materials [33]. Catalysts comprised of M-Nx species as active sites showed enhanced catalytic activity with lower HO_2_^−^ yield (less than 4) and a higher electron transfer number of 3.96 [33]. Furthermore, previous reports suggest that the ORR catalytic activity of the N-C catalysts is lower than that of Fe-N-C catalysts [31]. Since no Fe was detected by the XPS in GAL-Fe-N, the activity of GAL-Fe-N can be ascribed to the nitrogen species doped in the carbon matrix (N-C). Liu et al. [31] reported that the halfwave potential of the catalysts with N-C active sites shifts more negatively than that of Fe-N-C catalysts. The negative shift of the half wave potential and relatively high HO_2_^−^ yield confirms the active site of GAL-Fe-N is N-C. However, when compared to GAL-Fe-N, GAL-N showed lower electron transfer numbers of 2.97 to 3.33, and a significanly higher HO_2_^−^ (52.11–42.09%). The high electron transfer number and low HO_2_^−^ yield clearly demonstrated that the hybrid materials present in GAL-Fe-N (N-CNT and N-carbon spheres) provided more active sites for oxygen reduction than the N- carbon spheres present in GAL-N. Furthermore, the non-doped catalyst (GAL-Fe-A) provided different electron transfer activity from the N-doped GAL-Fe-N and GAL-N, showing a decrease in electron transfer number.

The enhanced catalytic activity on GAL-Fe-N can be attributed to the doped nitrogen in the graphitic framework, which altered the electro-neutrality of nanocarbons in both N-CNT and N-carbon spheres [34]. This significantly improved the catalytic activity for the ORR reaction by creating favourable charged sites for oxygen adsorption [8]. The pyridinic nitrogen structures (N is bound to the 2 nearest carbon atoms) with their strong Lewis basicity and electronic affinity in the N-CNT and N-carbon microspheres, induced a high positive charge density on the adjacent carbon atoms. As a result, the electron donor properties of nitrogen-doped (N-doped) GAL-Fe-N, triggered a favourable diatomic O–O adsorption weakening the O–O bond strength to facilitate ORR activity [35]. The recyclability of GAL-Fe-N was assessed by cycling the catalysts between 0.00 V and 1.15 V at 100 mV S^−1^ in an O_2_ saturated 0.1M KOH solution (SI). The result shows that after 6000 cycles the onset overpotential had increased 30 mV, showing only a slight deterioration of the catalysts.

**Figure 5:**
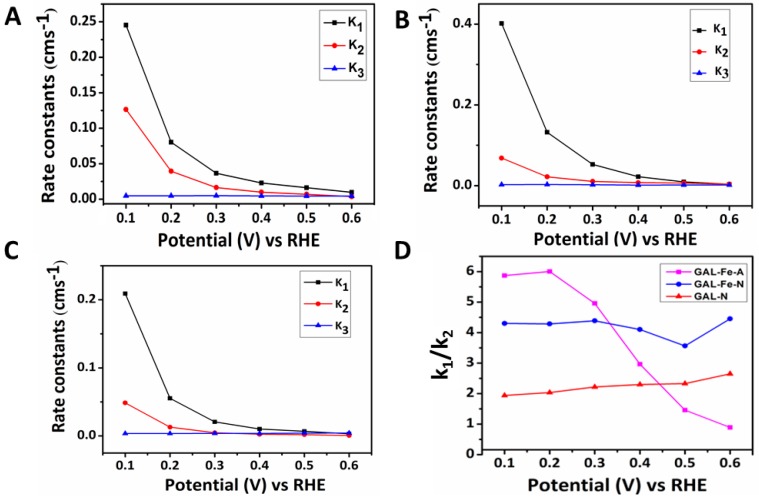
The rate constants for (**A**) GAL-N; (**B**) GAL-Fe-A; (**C**) GAL-Fe-Nis in the potential range of 0.1 V to 0.6 V; and (**D**) Comparison of k_1_/k_2_ ratio for GAL-N, GAL-Fe-A and GAL-Fe-N.

The electron transfer kinetics of ORR using RRDE were evaluated using the model proposed by Damjanovic et al. (SI.4) [36]. The materials’ rate constants were assessed within a range of 0.10–0.60 V and the results are displayed in Figure 5. For GAL-Fe-A, the k_1_/k_2_ ratio declined from 6.20 to 0.78, suggesting a two-electron transfer kinetic pathway as the potential increases. In contrast, the N-doped GAL-Fe-N (Figure 5D) is predominantly driven through four-electron kinetics, as the dissociation barrier of O_2_ on the carbon atoms adjacent to the N-doped material is reduced [37]. Although GAL-N showed k_1_/k_2_ > 1, the reaction produced a higher yield of H_2_O_2_. A comparison of the performance with the state-of-the-art catalysts (Table S1 in Supplementary Materials) shows that GAL-Fe-N is similar in activity. N-doped CNTs were not compared, as they could not be isolated from the hybridised material or synthesised independently using this method.

## 4. Conclusions

In summary, we have reported a new two-step process for the preparation of low-cost and environmentally friendly 3D N-doped carbon composite materials with integrated microsphere and nanotube morphologies. The use of melamine as a source of nitrogen and carbon for the formation of N-doped CNTs through the disruption of the surface on the carbon microspheres results from the release of FeMNPCs. The unique N-doped 3D composite prepared contains both N-doped microspheres and nanotubular structures, and functions as an ORR catalyst through a predominant four-electron transfer pathway.

## Figures and Tables

**Table 1:** Electrochemical properties of catalysts.

Catalyst	Onset Overpotential (V) RHE	Number of Electrons (n) (0.1–0.7 V) RHE	% HO_2_^−^ (0.1–0.7 V) RHE
GAL-N	0.33	2.97–3.33	52.11–42.09
GAL-Fe-A	0.45	3.66–2.75	16.39–62.31
GAL-Fe-N	0.29	3.55–3.64	22.44–16.96
Pt/C	0.26	3.71–3.97	11.67–9.59

## Supplementary Materials

The following are available online at www.mdpi.com/1996-1944/10/8/921/s1, Figure S1: EDX analysis on FeNP diffused out of sphere on GAL-Fe-N, Figure S2: SEM image of carbon sphere (A) GAL-Fe-A and (B) GAL-N, Figure S3: (A) Raman and (B) XRD spectra of GAL-Fe-A and GAL-Fe-N, Figure S4: (A) N_2_ adsorption/ desorption isotherm curve of (A) GAL-Fe-A and GAL-Fe-N and (B) pore size distribution of (B) GAL-Fe-A and (C) GAL-Fe-N, Figure S5: FTIR spectrum of (a) GAL-Fe-HT and (b) GAL-Fe-N, Figure S6: RDE polrarisation curves GAL-Fe-N with a scan rate of 10 mVs^−1^ before and after 6000 potential cycles in 0.1 M Oxygen saturated KOH, Figure S7: (A) XRD pattern of maghemite nanoparticles, Table S1: Comparison of the performance of GAL-Fe-N towards ORR with other similar carbon-based electro catalysts.
