# Supplementary material for: A Unique 3D Nitrogen-Doped Carbon Composite as High-Performance Oxygen Reduction Catalyst
Source: Materials (Basel). 2017 Aug 9;10(8):921. doi: 10.3390/ma10080921 (PMC5578287; doi:10.3390/ma10080921)
Supplement: Supplementary file 1 [file materials-10-00921-s001.pdf]

Supplementary Materials: A Unique 3D Nitrogen-Doped Carbon Composite as High-performance Oxygen Reduction Catalyst

Ramesh Karunagaran, Tran Thanh Tung, Cameron Shearer, Diana Tran, Campbell Coghlan, Christian Doonan[2], Dusan Losic

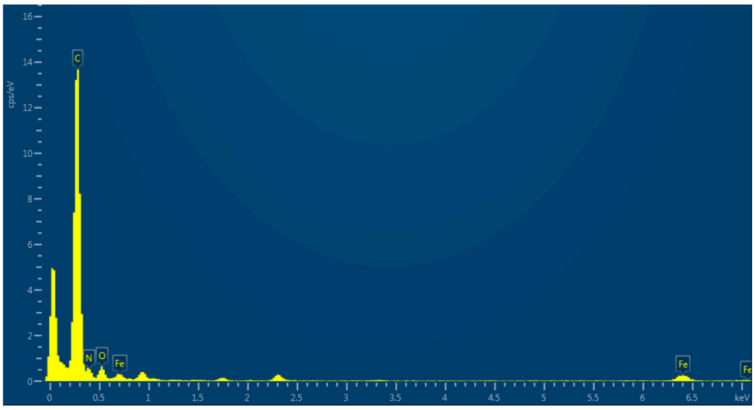

Figure S1 : EDX analysis on FeNP diffused out of sphere on GAL-Fe-N.

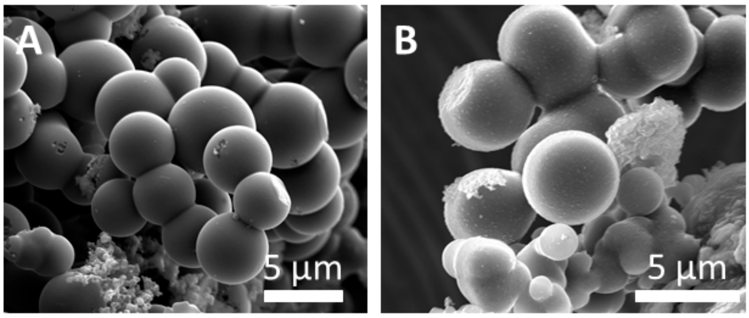

Figure S2 : SEM image of carbon sphere (A) GAL-Fe-A and (B) GAL-N

1. Raman and XRD characterisation

Raman spectroscopy performed on GAL-Fe-A and GAL-Fe-N (Figure S3A) showed characteristic peaks at 1338 cm^{-1} (D band) and 1585 cm^{-1} (G band) corresponds to the amorphous disordered carbon associated with the defects in the edges and vibrational bands of the highly ordered graphitic carbon domains, respectively [1,2]. The broad peak at 2684 cm^{-1} corresponding to the 2D peak indicates a few layers of the graphitic carbon structure [3-4]. The I_D/I_G ratio calculated to find the disorder associated with these samples showed 1.05 and 1.27 for GAL-Fe-A and GAL-Fe-N, respectively. Most notably the intensity ratio of the N-doped samples in the presence of iron (GAL-Fe-N), was higher than GAL-Fe-A. This can be ascribed to the incorporation of N and Fe in the graphene lattice which are distorting the hexagonally bonded carbon atoms in the graphitic framework and creating defects [5-6]. To determine the crystal structure of the prepared composites, XRD was performed on GAL-Fe-A and GAL-Fe-N and is shown in Figure S3B. The graph showed three characteristic peaks at 25.85, 42.21, 44.36 °C corresponding to (002), (110), and (101) facets, respectively for the amorphous graphitic carbon [7-8].

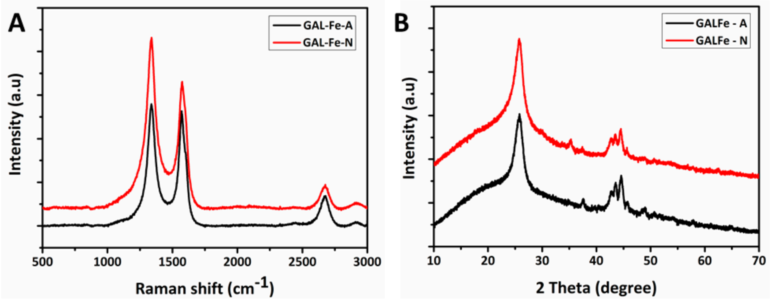

Figure S3: (A) Raman and (B) XRD spectra of GAL-Fe-A and GAL-Fe-N.

2. Surface area analysis of GAL-Fe-A and GAL-Fe-N

The nitrogen sorption isotherm and pore size distribution of GAL-Fe-A and GAL-Fe-N is shown in Fig. S4. The characteristic type IV isotherm and H4 hysteresis loop shows the presence of mesoporous slip-like pores [9]. The Barret-Joyner-Halenda (BJH) analysis measured a surface area of 311.03 and 101.76 m^2/g for GAL-Fe-A and GAL-Fe-N, respectively. Pore size distribution curves for GAL-Fe-A (Figure S4B) were calculated from the adsorption branch of the isotherm shows three different pores with mean pore sizes of 5.36 Å, 8.04 Å and 12.68 Å. The results show that the pores associated on the surface of the carbon microspheres contributed to the high surface area in GAL-Fe-A. However, the N-doping process disrupted the surface and interfered with the pores responsible for the higher adsorption and exposed the larger pores, therefore reducing the adsorption in GAL-Fe-N.

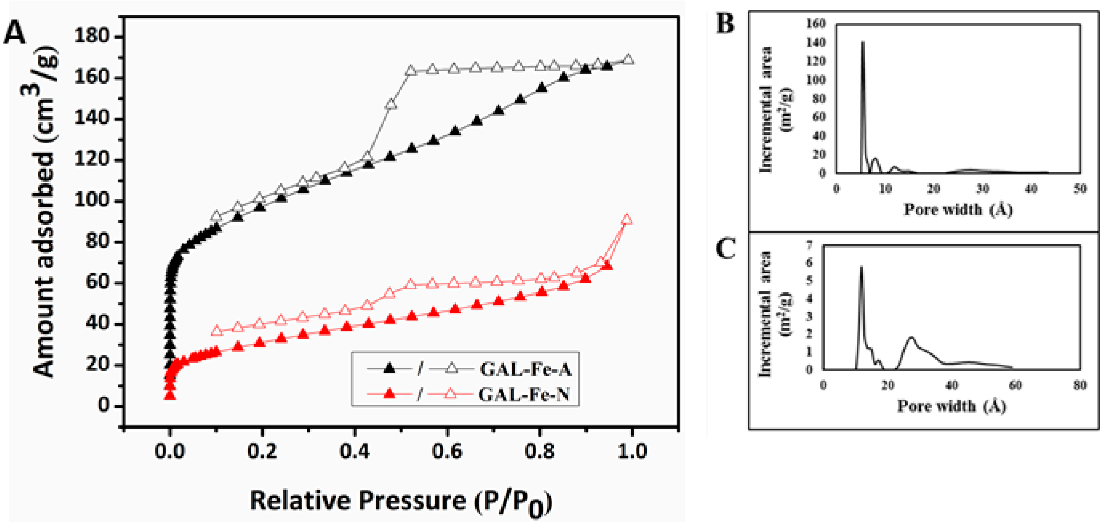

Figure S4: (A) N_2 adsorption/ desorption isotherm curve of (A) GAL-Fe-A and GAL-Fe-N and (B) pore size distribution of (B) GAL-Fe-A and (C) GAL-Fe-N.

3. FTIR analysis of GAL-Fe-HT and GAL-Fe-A.

FTIR analysis was conducted to analyse the functional groups present in the hydrothermally treated GAL-Fe-HT and N-doped GAL-Fe-N as presented in Fig. S5. The results show that GAL-Fe-HT had characteristic peaks at 1213, 1590, 1706 and 3390 cm^{-1} [10-12] which can be attributed to C-O and C-H stretching, stretching vibration of C=O, carbonyl vibrations and stretching vibration of O-H, respectively. The reduction of the intensities of these peaks in GAL-Fe-N (Figure S5b) can be attributed to the reduction of oxygen functional groups due to dehydration during the annealing process.

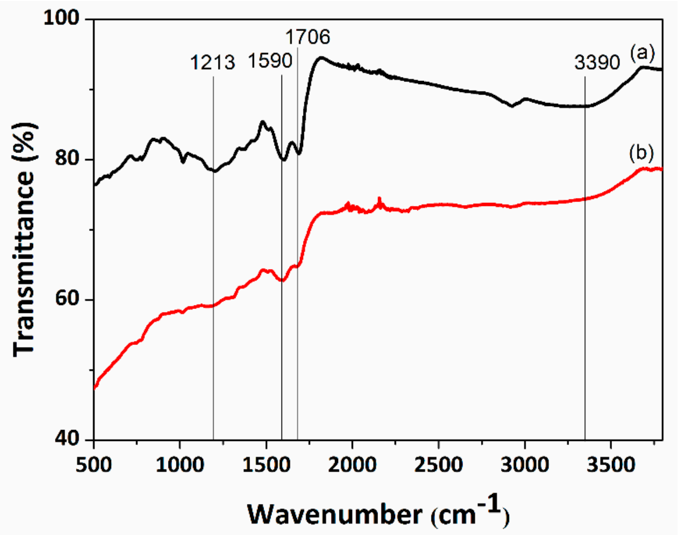

Figure S5 : FTIR spectrum of (a) GAL-Fe-HT and (b) GAL-Fe-N.

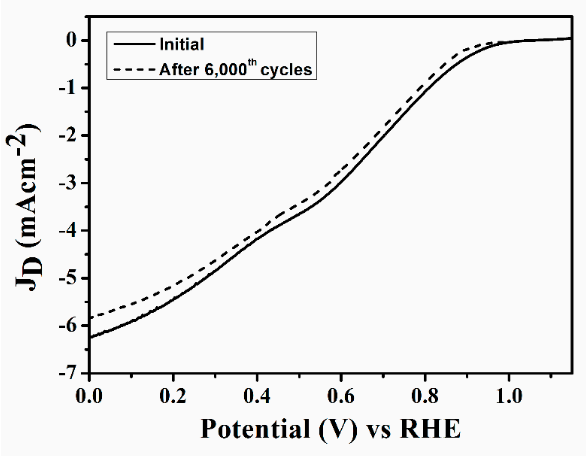

Figure S6: RDE polrarisation curves GAL-Fe-N with a scan rate of 10 mVs^{-1} before and after 6000 potential cycles in 0.1 M Oxygen saturated KOH.

4. Kinetics of electron transfer reaction of ORR.

Equation 1 and 2 were employed to further elaborate the dependance of number of electrons *(n)*and percentage of H_2O_2 with the applied potential.

$$n = \frac{4I_D}{I_D + \frac{I_R}{N}} \qquad (1)$$

$$\%H_2O_2 = 100\,\frac{2I_R}{I_D N + I_R} \qquad (2)$$

where I_D and I_R are the disc and ring currents, respectively and *N* is the collection efficiency [13].

To further study the kinetics of the ORR using RRDE voltammetry, Scheme 2 developed by Damjanovic et al. and Hsueh et al. was used for this study [14-15]. Damjanovic et al. [14] suggested that the mechanism of ORR should follow one of the two different pathways or a combination of both of them [14]. In other words, the reduction mechanism could follow a direct four electron pathway via k_1 kinetics and directly reduce oxygen to water or it could be driven through a two-electron parhway via k_2 kinetics producing peroxide intermediates and then be further reduced to water through another two-electron pathway through k_3 kinetics.

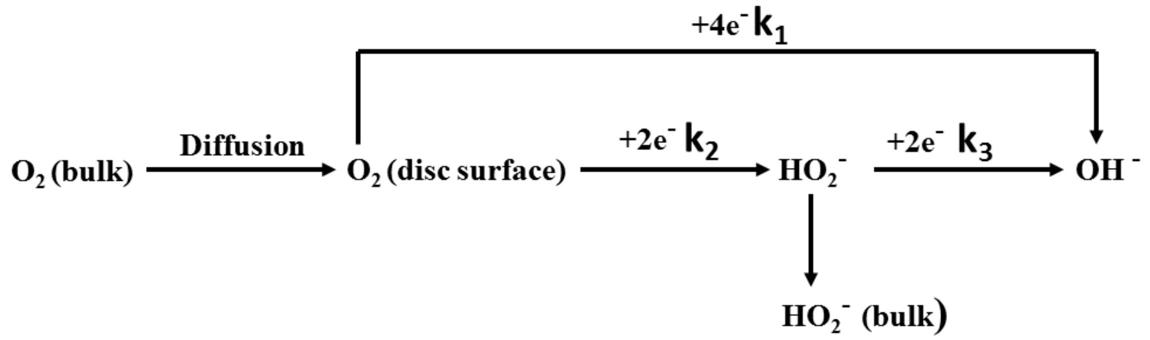

Scheme 2: Proposed model for electrochemical reduction of oxygen proposed by Damjanovic et al. and Hsueh et al.

A series of Equations (3, 4 and 5) suggested by Hsueh et al. [15] was used to calculate the rate constants k_1, k_2 and k_3. where Id, Ir, IdL and ω are the disc current, ring current, limiting disc current and the rotation speed, respectively. D_{O_2}, $D_{H_2O_2}$ and ν are the diffusion coefficient of oxygen, diffusion co efficient of H_2O_2 and kinematic viscosity, respectively. N is the collection efficiency.

$$k_1 = S_1 Z_1 \frac{I_1 N - 1}{I_1 N + 1} \quad (3)$$

$$k_2 = \frac{2\, S_2 Z_1}{I_1 N + 1} \quad (4)$$

$$k_3 = \frac{N S_1 Z_2}{I_1 N + 1} \quad (5)$$

Where S_1 and I_1 are the slope and intercept corresponding to I_d / I_r vs $\omega^{-1/2}$ plots and S_2 is the slope of I_{dL} / $I_{dL} - I_d$ vs $\omega^{-1/2}$ plot. $Z_1 = 0.62 D_{O_2}^{2/3} \nu^{-1/6}$, $Z_2 = 0.62 D_{H_2O_2}^{2/3} \nu^{-1/6}$, D_{O_2} is 1.4 x 10^{-5} cm^2 s^{-1}, $D_{H_2O_2}$ is 6.8 x 10^{-6} cm^2 s^{-1} and ν is 0.01 $cm^2 s^{-1}$ [16].

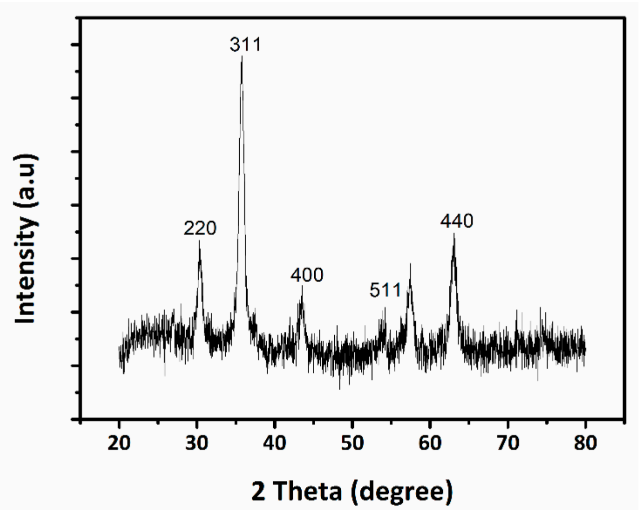

Figure S7: (A) XRD pattern of maghemite nanoparticles.

5. Mechanism of carbon sphere formation.

The water-soluble galactose can be attributed to the hydrophilicity of the resin solution due to the presence of oxygen containing functional groups formed from the sugars present in apricot sap. FeMNPs has the affinity to electrostatically bind to the negatively charged oxygen functional groups carbonaceous colloids [17]. During the polymerisation and carbonisation process of galactose, metal oxide nanoparticles can electrostatically attach to the oxygen functional groups and scattered as metal nanoparticles or metal clusters within the spheres. By collectively analysing the FTIR and Raman results of the annealed products, we can conclude the presence of hydrophilic oxygen groups and hydrophobic graphitic carbons present in the same product. It can be assumed that the mechanism

follows a similar pattern suggested by Sun *et.al* [18] using LaMer model [19] where the spheres were constructed with a hydrophilic shell and a hydrophobic core as shown in Scheme1.

Table S1: Comparison of the performance of GAL-Fe-N towards ORR with other similar carbon-based electro catalysts.

Material	Process	Sphere dimension	Onset over potential (V)	(n)/ Potential (V) RHE	Reference
N-doped mesoporous carbon spheres	Self-polymerisation	200 nm	0.37	3.40 / 0.575	(20)
N-doped hollow carbon spheres	Pyrolysis	400-700 nm	0.43	3.82 / 0.575	(21)
N-doped carbon spheres	Spray pyrolysis	130 – 500 nm	-	3.86/ 0.625	(22)
N-CNT	Pyrolysis	-	0.35	3.70 – 3.90/ 0.600 - 0.700	(23)
Integrated 3-d N-doped carbon sphere with CNT (GAL-Fe-N)	pyrolysis	1-6 μm	0.31	3.55 / 0.600	This study

6. References

1. V. Datsyuk, V.; Kalyva, M.; Papagelis, K.; Parthenios, J.; Tasis, D.; Siokou, A.; Kallitsis, I.; Galiotis, C. Chemical oxidation of multiwalled carbon nanotubes. Carbon. **2008**, 46, 833.
2. S. R. Ahmad, S. R.; Young, R. J.; Kinloch, I. A. Raman spectra and mechanical properties of graphene/polypropylene nanocomposites. Int. J. Chem. Eng.and Appl. **2015**, 6, 1.
3. Graf, D.; Molitor, F.; Ensslin, K.; Stampfer, C.; Jungen, A.; Hierold, C.; Wirtz, L. Spatially Resolved Raman Spectroscopy of Single- and Few-Layer Graphene. Nano Lett. **2007**, 7, 238.
4. Yoon, D.; Moon, H.; Cheong, H.; Choi, J. S.; Choi, J. A.; Park, B. H. *J.* Variations in the Raman Spectrum as a Function of the Number of Graphene Layers. Korean Phys. Soc. **2009**, 55, 1299.
5. Pimenta, M.; Dresselhaus, G.; Dresselhaus, M.S.; Cancado, L.; Jorio, A.; Saito, R. Studying disorder in graphite-based systems by Raman spectroscopy. Phys. Chem. Chem. Phys. **2007**, 9, 1276.
6. Schwan, J.; Ulrich, S.; Batori, V.; Ehrhardt, H.; Silva, S. Raman spectroscopy on amorphous carbon films. J. Appl. Phys. **1996**, 80, 440.
7. Suryawanshi, A.; Mhamane, D.; Nagane, S.; Patil, S.; Aravindan, V.; Ogale, S.; Srinivasan, M. Indanthrone derived disordered graphitic carbon as promising insertion anode for sodium ion battery with long cycle life. Electrochim. Acta. **2014**, *146*, 218.
8. Ma, X.; Yuan, C.; Liu, X. Mechanical, microstructure and surface characterizations of carbon fibers prepared from cellulose after liquefying and curing. Materials. **2013**, 7, 75.
9. Khalfaoui, M.; Knani, S.; Hachicha, M.; Lamine, A. B. New theoretical expressions for the five adsorption type isotherms classified by BET based on statistical physics treatment. J.Colloid Interface Sci. **2003**, 263, 350.
10. Elumalai, E. K.; Kayalvizhi, K.; Silvan, S. Coconut water assisted green synthesis of silver nanoparticles. J.Pharm. Bioallied Sci. **2014**, 6, 241.
11. Chen, J.; Xia, N.; Zhou, T.; Tan, S.; Jiang, F.; Yuan, D. Mesoporous carbon spheres: Synthesis, characterization and supercapacitance. Int. J. Electrochem. Sci. **2009**, 4, 1063.
12. Wang, H.; Hao,Q.; Yang, X.; Lu, L.; Wang, X. Graphene oxide doped polyaniline for supercapacitors. Electrochem.Commun. **2009**, 11, 1158.

13. Liang, Y.; Li, Y.; Wang, H.; Zhou, J.; Wang, J.; Regier, T.; Dai, H. Co3O4 nanocrystals on graphene as a synergistic catalyst for oxygen reduction reaction. Nat.Mater. **2011**, 10, 780.
14. Damjanovic, A.; Genshaw, M. A.; Bockris, J. O. Distinction between Intermediates Produced in Main and Side Electrodic Reactions. J. Chem. Phys. **1966**, 45, 4057.
15. Hsueh, K. L.; Chin, D. T.; Srinivasan, S. Electrode kinetics of oxygen reduction: A theoretical and experimental analysis of the rotating ring-disc electrode method. J. Electroanal. Chem. Interfacial Electrochem. **1983**, 153, 79.
16. Muthukrishnan, A.; Nabae, Y.; Chang, C. W.; Okajima, T.; Ohsaka, T. A high-performance Fe and nitrogen doped catalyst derived from diazoniapentaphene salt and phenolic resin mixture for oxygen reduction reaction. Catal. Sci. Tech. **2015**, 5, 1764.
17. Yu, G.; Sun, B.; Pei, Y.; Xie, S.; Yan, S.; Qiao, M.; Fan, K.; Zhang, X.; Zong, B. Fe x O y@ C spheres as an excellent catalyst for Fischer− Tropsch synthesis. J. Am. Chem. Soc. **2009**, 132, 935.
18. Sun, X.; Li, Y. Colloidal carbon spheres and their core/shell structures with noble-metal nanoparticles. Angew. Chem. Int. Ed. **2004**, 43, 597.
19. Mer, V. K. L. Nucleation in Phase Transitions. Ind. Eng. Chem. **1952**, 44, 1270.
20. J.Tang, J. Liu, C. Li, Y. Li, M.O. Tade, S. Dai and Y. Yamauchi, Synthesis of Nitrogen-Doped Mesoporous Carbon Spheres with Extra-Large Pores through Assembly of Diblock Copolymer Micelles. Angew. Chem. Int. Ed. **2015**, 54, 588.
21. Y.Li, T. Li, M.Yao and S. Liu, Metal-free nitrogen-doped hollow carbon spheres synthesized by thermal treatment of poly (o-phenylenediamine) for oxygen reduction reaction in direct methanol fuel cell applications. J. Mater. Chem. **2012**, *22* , 10911.
22. X. Zhou, Z. Yang, H. Nie, Z. Yao, L. Zhang and S. Huang, Catalyst-free growth of large scale nitrogen-doped carbon spheres as efficient electrocatalysts for oxygen reduction in alkaline medium. J. Power Sources. **2011**, 196 , 9970.
23. C. Xiong, Z. Wei, B. Hu, S. Chen, L. Li, L. Guo, W. Ding, X. Liu, W. Ji and X. Wang, Nitrogen-doped carbon nanotubes as catalysts for oxygen reduction reaction J. PowerSources. **2012**, 215, 216.
